# Treatment outcome and its predictors among patients of acute heart failure at a tertiary care hospital in Ethiopia: a prospective observational study

**DOI:** 10.1186/s12872-019-01318-x

**Published:** 2020-01-20

**Authors:** Mulubirhan Tirfe, Teshome Nedi, Desalew Mekonnen, Alemseged Beyene Berha

**Affiliations:** 1 Department of Pharmacy, College of Health Sciences and Comprehensive Specialized Hospital, Aksum University, Aksum, Ethiopia; 2 Department of Pharmacology and Clinical Pharmacy, School of Pharmacy , College of Health Sciences, Addis Ababa University, Churchill Avenue, P.O. Box 1176, Addis Ababa, Ethiopia; 3 Department of Internal Medicine, School of Medicine, College of Health Sciences, Addis Ababa University, Addis Ababa, Ethiopia

**Keywords:** Acute heart failure, In-hospital mortality, Predictor of mortality, Ethiopia

## Abstract

**Background:**

Acute heart failure is a rapid onset of new or worsening of signs and symptoms of heart failure that requires hospitalization or a visit to the emergency department. The aim of this study was to evaluate treatment outcome and determine factors that predict a poor treatment outcome in acute heart failure patients at a Tertiary Care Hospital in Ethiopia.

**Methods:**

A prospective observational study design was used. Data were collected using a structured questionnaire as a tool. Outcome variables were assessed at the time of discharge from the hospital. Bivariate and multivariate logistic regression analyses were used to determine factors that predict in-hospital mortality. A *p*-value ≤0.05 was considered as statistically significant.

**Results:**

Out of the 169 patients, the median age of patients with acute heart failure was 34 years (IQR = 23 to 50) and median hospital stay was 4.0 days (IQR = 3.0 to 6.0). The leading precipitating factor and underlying disease at the time of admission were pneumonia (47.5%) and chronic rheumatic heart disease (48.5%), respectively. The in-hospital mortality was found to be 17.2%. Smoking (adjusted odds ratio (AOR) = 8.7, *p* = 0.006), diabetes mellitus (AOR = 10.2, *p* = 0.005), pulmonary hypertension (AOR = 4.3, *p* = 0.016), and the presence of adverse drug events (AOR = 4.2, *p* = 0.003) were predictors of in-hospital mortality.

**Conclusion:**

High in-hospital mortality was observed among acute heart failure patients admitted to a Tertiary Care Hospital in Ethiopia. Smoking, diabetes mellitus, pulmonary hypertension and the presence of adverse drug events were predictors of in-hospital mortality.

## Background

Acute heart failure (AHF) is a rapid onset of new or worsening of signs and symptoms of heart failure (HF) that is associated with elevated plasma levels of natriuretic peptides [1]. AHF syndromes manifestas new-onset ‘de novo*’* or recurrence of acute decompensated heart failure (ADHF) requiring emergency treatment and hospitalization [2, 3]. The incidences of AHF vary in the different part of the world. Its increasing incidence is due to an increasing aging, population, complications arising from cardiovascular diseases like acute coronary syndrome (ACS) and increasing prevalence of lifestyle-related risk factors [4]. AHF patients who attended at hospitals in Africa are young and have severe symptoms due to late presentation. Thus, we should address the young people who are affected by the burden of an acute attack of HF. These younger age group had a significant impact on the economy of the society [5, 6].

Adherence to medication predicts health outcomes. Failure to adhere to HF medication was associated with poor treatment outcomes [7]. Patients should receive appropriate therapy as early as possible to achieve good treatment outcomes.

Evaluating reasons for hospitalization in AHF is important to give due attention to precipitating factors. The most common precipitating factors are non -compliance to salt restriction, pulmonary infections, arrhythmias and misuse of HF medications [8].The study conducted by Blecker S *et.al* has shown hospitalized AHF patients didn’t receive appropriate therapy [9]. Besides worsening, AHF was common in hospitalized patients and it was associated with higher mortality rates [10]. Therefore, managing AHF patients according to guideline recommendation could reduce patient hospitalization, decrease morbidity and mortality.

Limited studies and literatures are available in Africa and other developing countries that describe the clinical characteristics, management, and treatment outcome of AHF patients. Therefore, the present study could provide valuable insights to the patient’s treatment outcome and predictors of in- hospital mortality among patients hospitalized with AHF in Ethiopia.

## Methods

### Study design and setting

A hospital-based prospective observational study design was used. The study was conducted from May 15 to September 12, 2017, through a structured data abstraction tool. This study was conducted at Tikur Anbessa Specialized Hospital, Addis Ababa, Ethiopia. Tikur Anbessa Specialized Hospital is the largest referral hospital in the country. It has over 700 beds, and serves about 310,000 and 32,000 patients per year in its outpatient and inpatient departments, respectively. The emergency department (ED) also provides services to about 29,000 patients per year and on average 50 patients per day [11]. All patients admitted to the hospital with a diagnosis of AHF during the study period were recruited.

### Data collection

The data abstraction tool included socio-demographic characteristics, clinical features, laboratory data, precipitating factors, underlying diseases, co-morbidity, imaging studies, treatments given and hospital stay. The treatment outcome was assessed at the time of discharge from the hospital.

### Ethical clearance

Ethical clearance was obtained from the Ethical Review Committee of School of Pharmacy, College of Health Sciences, Addis Ababa University (Ref. no ERB/SOP/20/09/2017). Permission was also obtained from the Department of Internal Medicine, School of Medicine, College of Health Sciences, Addis Ababa University. Informed oral consent was obtained from patients and for those whose age was < 18 years consent as well as assent was obtained from guardians.

### Data analysis

Findings were presented as mean ± (SD) for normal distributed, otherwise median (inter-quartile range) for non-normal distributed variables. Categorical variables were reported as percentages and frequency Tables. A chi-square test was used for categorical variables. Bivariate and multivariate logistic regression was used to analyze factors that predict poor treatment outcomes, and variables whose *p*-values < 0.2 in the univariate analysis were included in the multivariate model. The level of significance was chosen at p-value ≤0.05 and results were reported as 95% confidence intervals. For all statistical analysis Statistical Package for Social Sciences (SPSS version 20) was used.

### Data quality assurance

One day training was given for data collectors on the importance, objectives, and method of data collection. There was on-going supervision by the principal investigator. A pre-test was done on 11 consecutive patients to assure clarity, avoidance of ambiguity, comprehensiveness and content uniformity.

### Operational definitions

**Acute heart failure: -** sign and symptoms of new-onset of HF and/or decompensation or worsening of chronic stable HF; **Adverse drug events: -** any injury occurring during the patient’s drug therapy and resulting either from appropriate care or unsuitable or suboptimal care; **Evidence-based guidelines: -** consensus approaches for handling recurring health management problems aimed at reducing practice variability and improving health outcomes. **Smoker:-** those who are current smokers and had a history of smoking in the last 1 month only; **Inappropriate dose:-** defined according to European Society of Cardiology management of AHF in the first 48 h used as a reference [1].

## Results

### Socio-demographic characteristics

From a total of 181 AHF patients admitted to the emergency and medical wards of Tikur Anbessa Specialized Hospital between May 15 to September 12, 2017; 12 patients declined to participate and a total of 169 patients who were diagnosed with AHF were included in the study. Of these, 120 (71.0%) patients are urban residents; 104 (61.5%) were married; and 92 (54.4%) of the patients were females. Of the 169 patients, nine (5.3%) were smokers and 16 (9.5%) of the patients were readmitted during the study period (Table 1).

**Table 1:** Socio-demographic characteristics of acute heart failure patients admitted to Tikur Anbessa Specialized Hospital, Ethiopia between May 15 to September 12, 2017 (*n* = 169)

Variable	Description	Frequency (%)
Residence	Urban	120 (71.0)
	Rural	49 (29.0)
Education	No formal education	30 (17.8)
	Primary school	71 (42.0)
	Secondary school	30 (17.8)
	Higher education	38 (22.5)
Marital status	Married	104 (61.5)
	Single	54 (32.0)
	Divorced	7 (4.2)
	Widowed	4 (2.4)
Gender	Female	92 (54.4)
	Male	77 (45.6)
Smoking	Yes	9 (5.3)
	No	160 (94.7)
Alcohol intake	Yes	15 (8.9)
	No	154 (91.1)
Readmission	Yes	16 (9.5)
	No	153 (90.5)

As shown in Table 2, the median age of patients was 34.0 years (interquartile range (IQR) = 23 to 50). The median time required to reach the health institutions was 1:00 h (IQR = 0:30 to 2:00). The median hospital stay of patients was 4.0 days (IQR = 3.0 to 6.0).

**Table 2:** Vital signs and ejection fraction of acute heart failure patients admitted to Tikur Anbessa Specialized Hospital, Ethiopia between May 15 to September 12, 2017 (*n* = 169)

Variables	N	Frequency (%)	Mean	SD	Median	Q1	Q3
Age (years)	169		37.8	17.8	34.0	23.0	50.0
Time required to reach HI (hr)	169		1:17	0:55	1:00	0:30	2:00
Hospital stay (days)	169		7.1	10.0	4.0	3.0	6.0
Ejection fraction (%)	74		53.8	13.7	55.0	45.8	63.3
Preserved EF (≥ 50%)		55 (74.3)					
Reduced EF (≤ 40%)		15 (20.3)					
Mid-range EF (41 to 49%)		4 (5.4)					

Key: *HI* health institution, *SD* standard deviation, Q1. = 25th percentile, Q3 = 75th percentile

### Clinical characteristics

Out of the total 169 AHF patients, 74 (44.0%) of them had a documented echocardiographic measurement with the result of 55 (74.3%) had preserved ejection fraction and 15 (20.3%) had reduced ejection fraction. In accordance with Framingham’s heart failure criteria, 99 (59.0%) had a combination of two or more Framingham major criteria at admission. Among these most frequently observed combinations of major criteria were neck vein distension and paroxysmal nocturnal dyspnea, followed by neck vein distension and S_3_ gallop (Table 3).

**Table 3:** Framingham major criteria of acute heart failure patients admitted to Tikur Anbessa Specialized Hospital, Ethiopia between May 15 to September 12, 2017 (*n* = 169)

Combination Framingham major criteria	Frequency (%)
Neck vein distension + Paroxysmal nocturnal dyspnea	30 (17.8)
Neck vein distension + S_3_ gallop	12 (7.1)
Neck vein distension + Rales	10 (5.9)
Paroxysmal nocturnal dyspnea + S_3_ gallop	10 (5.9)
Neck vein distension + Acute pulmonary edema	6 (3.6)
Paroxysmal nocturnal dyspnea + Rales	6 (3.6)
Neck vein distension + Paroxysmal nocturnal dyspnea + Rales	5 (3.0)
Neck vein distension + Paroxysmal nocturnal dyspnea + Acute pulmonary edema	4 (2.4)
Paroxysmal nocturnal dyspnea + Acute pulmonary edema	4 (2.4)
Neck vein distension + Rales + S_3_ gallop	4 (2.4)
Other combination criteria^a^	8 (4.8)

NB: ^a^(Neck vein distension + Acute pulmonary edema + S_3_ gallop), (Acute pulmonary edema + S_3_ gallop), (Acute pulmonary edema + Cardiomegally), (Neck vein distension + Paroxysmal nocturnal dyspnea + S_3_ gallop), (Rales + S_3_ gallop), (Acute pulmonary edema + Rales + S_3_ gallop)

Serum potassium was determined during presentation for 116 patients with mean ± (SD) value of 4.1 ± 0.9 mEq/L; of these, 32 (27.6%) had serum potassium concentration <  3.55 mEq/L, and 7(6.0%) had serum potassium concentration >  5.55 mEq/L. The mean ± (SD) value of hemoglobin for 158 patients at admission was 12.8 ± 3.0 g/dL, among those 24 (15.2%) had clinically significant anemia (hemoglobin < 10 g/dL).

Blood urea nitrogen and serum creatinine measurement was found for 62 and 145 of the patients, respectively; median value of BUN was 70.6 mg/dL (IQR = 49.2, 127.3) and 47 (75.8%) had elevated BUN level (≥ 43 mg/dL). Elevated serum creatinine value (≥ 1.2 mg/dL) was found in 58 (40.0%) of the patients at admission.

Data for estimated glomerular filtration rate (GFR) was found for 145 patients with mean ± (SD) value of 74.2 ± 33.0 mL/min/1.73m^2^. Of the 145 patients based on Modification of Diet in Renal Disease (MDRD) derived formula 12 (8.3%) patients had ≤30 mL/min/1.73m^2^ value, 30 (20.7%) patients had 30 to 59 mL/min/1.73m^2^ and 61 (42.1%) patients had 60 to 89 mL/min/1.73m^2^ estimated GFR (See in Table 4).

**Table 4:** Laboratory values of acute heart failure patients admitted to Tikur Anbessa Specialized Hospital, Ethiopia between May 15 to September 12, 2017 (*n* = 169)

Variables	N	Frequency (%)	Mean	SD	Median	Q1	Q3
Serum sodium (mEq/L)	120		135.8	10.1	136.5	130.5	143.3
Serum sodium ≥135 mEq/L		72 (60.0)					
Serum sodium < 135 mEq/L		48 (40.0)					
Serum potassium (mEq/L)	116		4.1	0.9	4.0	3.47	4.53
Normal		77 (66.4)					
Hypokalemia (< 3.55 mEq/L)		32 (27.6)					
Hyperkalemia (> 5.55 mEq/L)		7 (6.0)					
Hemoglobin (g/dL)^a^	158		12.8	3.0	12.8	10.8	14.8
Hemoglobin ≥12 g/dL		98 (62.0)					
Hemoglobin < 12 g/dL		60 (38.0)					
Hemoglobin < 10 g/dL		24 (15.2)					
Serum creatinine (mg/dL)	145		1.6	1.8	1.2	1.0	1.5
Normal serum creatinine ≤1.2		87 (60.0)					
Elevated serum creatinine > 1.2		58 (40.0)					
Blood urea nitrogen (mg/dL)	62		110.5	95.3	70.6	49.2	127.3
Less than 43 mg/dL		15 (24.2)					
Elevated ≥43 mg/dL		47 (75.8)					
eGFR (mL/min/1.73m^2^)	145		74.2	33.0	74.0	55.0	92.0
≤14		8 (5.5)					
15 to 29		4 (2.8)					
30 to 59		30 (20.7)					
60 to 89		61 (42.1)					
≥90		42 (29.0)					

NB: ^a^Measurement taken after patient was stabilized Key: eGFR = estimated glomerular filtration rate, SD = standard deviation; Q1 = 25th percentile, Q3 = 75th percentile

Findings for chest X-ray, electrocardiogram (ECG) and echocardiography were obtained for 42 (25.0%), 109 (64.5%) and 83 (49.1%) patients, respectively. Of the 109 patients who had ECG, atrial fibrillation was detected in 59 (54.1%) of the patients. (See in Table 5).

**Table 5:** Imaging findings of acute heart failure patients admitted to Tikur Anbessa Specialized Hospital, Ethiopia between May 15 to September 12, 2017 (*n* = 169)

Imaging	Finding	Frequency (%)
Chest X-ray (*N* = 42)	Pulmonary edema	27 (64.3)
	Pleural effusion	12 (28.6)
	Pneumonia	6 (14.3)
	Normal	3 (7.1)
Electrocardiogram (*N* = 109)	Atrial fibrillation	59 (54.1)
	Sinus tachycardia	37 (33.9)
	Sinus rhythm	12 (11.0)
	Bradycardia	5 (4.6)
Echocardiography (*N* = 83)	Chronic rheumatic heart disease	67 (80.7)
	Pulmonary hypertension	55 (66.3)
	Hypertensive heart disease / LVH	11 (13.3)
	Ischemic heart disease	8 (9.6)
	Pericardial effusion	3 (3.6)
	Others (normal, degenerative heart disease)	4 (2.4)

Key: *LVH* Left ventricular hypertrophy

Factors that precipitate AHF at admission were found for 160 patients, among these the top four precipitating factors were pneumonia 76 (47.5%), atrial fibrillation 55 (34.4%), anemia 39 (24.4%), and drug discontinuation 36 (22.5%). The three most common underlying diseases found in AHF patients in order of decreasing frequency were chronic rheumatic heart disease (RHD) 82 (48.5%), degenerative valvular heart disease (VHD) 37 (22.5%), and congenital heart disease (CHD) 33 (19.5%). Chronic kidney disease (CKD) 18 (10.7%) was the most common co-morbid disease followed by pulmonary hypertension 17 (10.1%) and hypertension 14 (8.3%) (See in Table 6).

**Table 6:** Precipitating factors, underlying and co-morbid diseases of acute heart failure patients admitted to Tikur Anbessa Specialized Hospital, Ethiopia between May 15 to September 12, 2017 (*n* = 169)

Factors / diseases	Frequency (%)
Precipitating factors (*n* = 160)	
Pneumonia	76 (47.5)
Atrial fibrillation	55 (34.4)
Anemia	39 (24.4)
Drug discontinuation	36 (22.5)
Infective endocarditis	7 (4.4)
Acute coronary syndrome	6 (3.8)
Others (Pregnancy, uncontrolled hypertension)	6 (3.8)
Underlying (*n* = 169)	
Chronic rheumatic heart disease	82 (48.5)
Degenerative heart disease	38 (22.5)
Congenital heart disease	33 (19.5)
Hypertensive heart disease	17 (10.1)
Ischemic heart disease	17 (10.1)
Cor pulmonale	14 (8.3)
Dilated cardiomyopathy	14 (8.3)
Co-morbid (*n* = 169)	
Chronic kidney disease	18 (10.7)
Pulmonary hypertension	17 (10.1)
Hypertension	14 (8.3)
Diabetes mellitus	8 (4.7)
Coronary artery disease	8 (4.7)
Asthma	8 (4.7)
Chronic obstructive pulmonary disease	7 (4.1)
Tuberculosis	6 (3.6)
HIV/AIDS	4 (2.4)
Others^a^	8 (4.7)

NB: ^a^Cancer, hyperthyriodism, pericardial effusion, stroke

### Treatment outcome and its predictors

Out of the total 169 patients, 140 (82.8%) patients had clinically improved and 29 (17.2%) patients died while they were at the hospital. Adverse drug events were found in 47 (27.8%) of the patients and inappropriate drug dose was prescribed to 5(3.0%) patients (Table 7).

**Table 7:** Treatment outcome and drug use assessment of acute heart failure patients admitted to Tikur Anbessa Specialized Hospital, Ethiopia between May 15 to September 12, 2017 (*n* = 169)

Treatment outcome and drug use assessment	Frequency (%)
Clinically improved	140 (82.8)
In-hospital mortality	29 (17.2)
Adverse drug events	47 (27.8)
Electrolyte imbalance	39 (23.1)
Digoxin and warfarin toxicity	8 (4.7)
Inappropriate drug dose	5 (3.0)
Others^a^	4 (2.4)

NB: ^a^Inappropriate drug administered, inappropriate drug combination, prescribed drug not available

In the chi-squared test, smoking, pulmonary hypertension, diabetes mellitus and presence of adverse drug events were independently associated with in-hospital mortality of AHF (See in Table 8).

**Table 8:** Chi-square test of variables associated with in-hospital mortality of acute heart failure patients admitted to Tikur Anbessa Specialized Hospital, Ethiopia between May 15 to September 12, 2017 (*n* = 169)

Variables	Percent poor treatment outcome (95% CI)	*P* value
Adverse drug events(ADR/SE) absent	12.3% (6.4, 18.2)	
Adverse drug events(ADR/SE) present	29.8% (16.7, 42.9)	0.007
One Framingham major criterion	13.8% (13.4, 14.2)	
More than one Framingham major criteria	22.1% (14.1, 30.1)	0.041
Diabetes mellitus absent	15.6% (10.4, 21.8)	
Diabetes mellitus present	50.0% (15.3, 84.7)	0.031
Pulmonary hypertension absent	15.1% (9.4, 20.8)	
Pulmonary hypertension present	41.2% (17.9, 64.5)	0.013
Non-smokers	15.6% (9.9, 21.3)	
Smokers	44.4% (11.9, 76.9)	0.048

Key: *ADR/SE* Adverse drug reaction/side effect, *CI* confidence interval

In multivariate logistic regression analysis smoking, diabetes mellitus, pulmonary hypertension and presence of adverse drug events had a statistically significant association with in-hospital mortality (See in Table 9).

**Table 9:** Univariate and multivariate logistic regression analysis of acute heart failure patients admitted to Tikur Anbessa Specialized Hospital, Ethiopia between May 15 to September 12, 2017 (*n* = 169)

Variables	Univariate		*P* value	Multivariate		*P* value
	OR	95% CI		AOR	95% CI	
Socio-demographic						
Age ^b^	1.0	0.98, 1.00	0.89			
Alcohol intake ^a^	1.9	0.55, 6.40	0.30			
Salt intake ^a^	0.7	0.23, 1.80	0.40			
Smoking ^a^	4.3	1.10, 17.20	0.038	8.72	1.84, 41.30	0.006
Diseases						
Atrial fibrillation ^a^	1.6	0.67, 3.70	0.30			
Cancer ^a^	10.3	0.9, 117.6	0.06			
Chronic kidney disease ^a^	1.4	0.40, 4.70	0.56			
Chronic rheumatic heart disease ^a^	1.9	0.86, 4.40	0.11			
Diabetes mellitus ^a^	5.4	1.30, 23.20	0.022	10.18	2.04, 50.85	0.005
Hypertension ^a^	1.4	0.40, 5.20	0.67			
Hypertensive heart disease ^a^	2.2	0.70, 6.90	0.17			
Pneumonia ^a^	1.3	0.60, 3.00	0.48			
Pulmonary hypertension ^a^	4.1	1.40, 12.00	0.009	4.33	1.31, 14.25	0.016
Other						
Adverse drug events ^a^	3.0	1.30, 6.90	0.009	4.23	1.62, 11.02	0.003
Echocardiography finding*	3.2	0.66, 15.50	0.15			
Framingham major criteria**	2.7	1.00, 6.90	0.047	2.89	0.98, 8.50	0.054

Key: *AOR* adjusted odds ratio, *CI* confidence interval

NB: (*) coded 1 = one major criteria, 2 = two or more major criteria, (**) coded 1 = one finding, 2 = more than one finding; (a) coded 0 = no, 1 = yes; (b) for 1 unit increase; serum Na coded 1 > 135, 2 < 135; systolic blood pressure = coded 1 ≥ 115, 2 < 115; serum creatinine coded 1 ≤ 1.2 and 2 > 1.2

## Discussion

AHF patients presenting to a tertiary care hospital were young and had pneumonia as a major precipitating factor. The leading underlying disease was chronic rheumatic heart disease (RHD) and major co-morbid disease was chronic kidney disease (CKD). 17.2% of the patients had died in the hospital.

More than half of the AHF patients in this study were female 92 (54.4%) which was comparable to registry studies in sub-Saharan Africa Survey on Heart Failure (50.8%), the 5 year retrospective cohort study of African patients admitted with heart failure (54.4%), Acute Decompensated Heart Failure Registry (ADHERE) (52.0%) and the Organized Program to Initiate Lifesaving Treatment in Hospitalized Patients with Heart Failure (OPTIMIZE-HF) (52.0%) [5, 12–14]. However, the European registries Euro Heart Failure Survey II (EHFS II) (39.0%), Heart Failure Pilot Study (ESC-HF pilot) (47.0%) and the Acute Heart Failure Global Registry of Standard Treatment (ALARM-HF) (37.6) studies females had lower frequency as compared to males [15–18].

Patients admitted in this study were young (median = 34 years). This was contrary to registries in ADHERE (mean = 72.4), OPTIMIZE-HF (mean = 73), OFICA (median = 79.0), Korean Acute Heart Failure Registry (KorAHF) (mean = 68.5) and the sub-Saharan Africa Survey of Heart Failure cohort (mean = 52.3) [5, 12, 15, 19, 20]. Reason for younger age admission could be related to the high prevalence of RHD in Ethiopia [21]. Similarly, in the study by Abdissa and his colleagues the peak age of diagnosis with VHD among Ethiopian patients was in their third decade mean ± (SD) = 24.4 ± 9.7 years [22]. AHF in Tikur Anbessa Specialized Hospital pediatric ward was primarily due to RHD [21]. Supported by Soweto study 2006/07 South Africa, RHD was peaked predominantly in the third decade of life [23].

In the present study leading precipitating factors were pneumonia, atrial fibrillation, anemia and drug discontinuation. Similar to our study, in the OPTIMIZE-HF registry study pneumonia, ischemia/acute coronary syndrome and arrhythmia were leading precipitating factors [13].. This was also comparable with the ALARM-HF registry study where arrhythmia, infection and non-compliance to medication were the most frequent precipitating factors [18].

In the current study drug discontinuation as precipitating factor was reported in 22.5% of the patients, and almost 101 (59.8%) patients had a primary school and no formal education. Adherence to heart failure medication regimens could be influenced by inadequate support, lack of education and illiteracy. In addition to optimal pharmacologic treatment patient education on medication adherence had improved outcomes, [2]. Thus, clinical pharmacists had key role in medication adherence of heart failure patients as demonstrated by the Pharmacist in Heart Failure Assessment Recommendation and Monitoring (PHARM) study. Interventions made by clinical pharmacists lowered readmission/death by more than 50.0% through closer follow-up [24].

The leading underlying disease found was chronic RHD this was supported by different studies. In developing countries heart failure was primarily due to VHD whereas developed countries it was mainly due to ischemic disease [25]. Studies in the African population showed RHD was the commonest diagnosis among patients with cardiovascular diseases. The study at Jimma Specialized Hospital in Ethiopia showed RHD was the commonest diagnosis among patients presented to the cardiac clinic [26]. In Tikur Anbessa Specialized Hospital, the VHD was the commonest diagnosis among patients with cardiovascular diseases [22]. This was also similar to studies done in sub-Saharan Africa where cardiomyopathy and RHD accounted for almost half of all cases presented to hospitals [27–29]. However, this was different from THESUS–HF study where hypertensive and ischemic heart disease(IHD) were the primary causes [5].

This study showed that treatment was targeted mainly towards symptom relief, the underlying and/or co-morbid disease were most commonly treated by frusemide, spironolactone, digoxin and warfarin. In the current study, warfarin and digoxin had higher consumption rates which were used for the management of VHD and atrial fibrillation [2, 30, 31].

The present study showed in-hospital mortality of 17.2% which is comparable with the mortality of THESUS–HF cohort study [5]. This was higher than the study of systematic review and meta-analysis in low and middle income countries that a 2.2% of hospital admission was AHF with mean in-hospital mortality of 8% [32].Globally, hospitalized patients had higher mortality (30.6%) and African patients had the highest adjusted hazard of death (34%) within 1 year. This variation in mortality might be related to the difference in health-care infrastructure, quality of care, environmental and genetic factors in different regions of the world [33].

Adverse drug events were a predictor of in-hospital mortality that occurred in 27.8% of the patients. This study found hypokalemia in 27.6% and hyperkalemia in 6.0% of the patients. Use of loop diuretic could lead to hypokalemia and drugs that increase potassium level especially in renal dysfunction such as a combination of angiotensin converting enzyme inhibitors, potassium chloride and spironolactone could lead to hyperkalemia. Use of non-potassium sparing diuretics was significantly associated with increased risk of arrhythmic death. Diuretic-induced electrolyte disturbance might ultimately resulted in fatal arrhythmia in patients using non-potassium sparing diuretics [34].

In our study AHF patients with pulmonary hypertension had higher mortality. Similarly, Lowe and colleagues showed patients with pulmonary hypertension had twofold risk of mortality [35]. The increased mortality in patients with pulmonary hypertension might be due to an aggressive afterload reduction with vasodilator or diuretics treatment that could finally end up in cardiovascular collapse as these patients could not increase their forward blood through flow restricted valve [36].

Patients with heart failure and preserved (≥ 50%) ejection fraction have multiple co-morbidities including diabetes mellitus, atherosclerosis, renal dysfunction, chronic obstructive lung disease, and anemia. The presence of those co-morbidities were associated with increased all-cause mortality among patients [14].The co-morbidities of heart failure patients (preserved EF) were significantly associated with unique clinical, structural, functional and prognostic profiles [37]. The poor clinical outcome of heart failure patients with preserved ejection fraction can not merely be explained by age, sex, presence of co-morbidity, low blood pressure and left ventricular remodeling rather than additional involvement of heart failure related mechanisms explained the worse outcome of patients [38]. The variation in cardiac and non-cardiac co-morbid conditions, underlying diseases, and clinical profile at presentation, diagnostic and treatment in AHF were heterogeneous across different countries [5, 12, 13, 15, 16, 39].

There are limited studies on the causes, treatments and outcomes of AHF in Africa. The present study provides information on the clinical characteristics, underlying and co-morbid disease, adverse drug events and treatment outcome and its predictors in AHF in Ethiopia. Ultimately the findings are useful for the policymaker to develop strategies to improve treatment outcomes, quality of care, preventive and diagnostic services of AHF.

### Limitations

The present study has the following limitations. The study was conducted in a single -center with a small sample size. In addition, measurements on biomarkers and laboratory values like BNP, NTproBNP, high sensitive C-reactive protein and uric acid were not available in this study that could be used significantly to predict the outcome of AHF. Besides measurement on cardiac troponin, creatine kinase–MB and BUN were not obtained fully.

## Conclusion

High in-hospital mortality rate (17.2%) was observed among acute heart failure patients admitted to a Tertiary Care Hospital in Ethiopia. Chronic RHD and pneumonia were the leading underlying disease and precipitating factors found in patients admitted with AHF, respectively. Smoking, diabetes mellitus, pulmonary hypertension and the presence of adverse drug events were predictors of poor treatment outcomes. Due attention should be given to co-morbid diseases while patients presented with AHF syndromes. Clinicians should also pay more attention to the management of adverse drug events.

## Data Availability

The datasets used and/or analyzed during the current study are available from the corresponding author on reasonable request.

## Abbreviations

ACC/AHA: American College of Cardiology / American Heart Association
ACE: Angiotensin converting enzyme
ACS: Acute coronary syndrome
ADHERE: Acute decompensated heart failure National Registry
ADHF: Acute decompensated heart failure
ADR/SE: Adverse drug event / side effect
AF: Atrial fibrillation
AHF: Acute heart failure
ALARM-HF: Acute heart failure global registry of standard treatment
BNP: B-type natriuretic peptide
BUN: Blood urea nitrogen
CHD: Congenital heart disease
CKD: Chronic kidney disease
DM: Diabetes mellitus
ED: Emergency department
EF: Ejection fraction
EHFS II: EuroHeart failure survey II
ESC: European society of cardiology
ESC-HF pilot: EURObservational research program the heart failure pilot survey
GFR: Glomerular filtration rate
HF: Heart failure
IHD: Ischemic heart disease
KorAHF: Korean acute heart failure registry
MDRD: Modification of diet on renal disease
MI: Myocardial infarction
NTproBNP: N-terminal pro-B-type natriuretic peptide
NYHA: New York heart association
OPTIMIZE-HF: Organized program to initiate lifesaving treatment in hospitalized patients with heart failure
RHD: Rheumatic heart disease
SBP: Systolic blood pressure
THESUS–HF: The sub-Saharan Africa survey of heart failure
VHD: Valvular heart disease
